# Epileptic Status in a PEDiatric cohort (ESPED) requiring intensive care treatment: A multicenter, national, two‐year prospective surveillance study

**DOI:** 10.1002/epi4.12707

**Published:** 2023-02-20

**Authors:** Sascha Meyer, Jaro Langer, Martin Poryo, Johannes Goaliath Bay, Stefan Wagenpfeil, Beate Heinrich, Holger Nunold, Adam Strzelczyk, Daniel Ebrahimi‐Fakhari

**Affiliations:** ^1^ Department of General Pediatrics and Neonatology, and Neuropediatrics Saarland University Medical Center Homburg Germany; ^2^ Franz‐Lust Klinik für Kinder und Jugendliche Karlsruhe Germany; ^3^ Department of Pediatric Cardiology and Pediatric Intensive Care Medicine Saarland University Medical Center Homburg Germany; ^4^ Institute for Medical Biometry, Epidemiology and Medical Informatics Saarland University Homburg Germany; ^5^ Erhebungseinheit für Seltene Pädiatrische Erkrankungen (ESPED) Heinrich‐Heine University Düsseldorf Düsseldorf Germany; ^6^ Epilepsy Center Frankfurt Rhine‐Main, Center of Neurology and Neurosurgery Goethe‐University and University Hospital Frankfurt Frankfurt am Main Germany; ^7^ Center for Personalized Translational Epilepsy Research (CePTER) Goethe‐University Frankfurt Frankfurt am Main Germany; ^8^ Department of General Pediatrics University Children's Hospital Muenster Muenster Germany

**Keywords:** anti‐seizure medication., diagnosis, follow‐up, intensive care treatment, management, pediatric, seizure, status epilepticus, surveillance, therapy

## Abstract

**Objective:**

The aim of this study was to provide seizure etiology, semiology, underlying conditions, and out‐of‐ and in‐hospital diagnostics, treatment, and outcome data on children with out‐of‐ or in‐hospital‐onset status epilepticus (SE) according to the International League Against Epilepsy definition that required admission to the pediatric intensive care unit (PICU) for ≥4 hours.

**Methods:**

This prospective national surveillance study on SE in childhood and adolescence was conducted over 2 years (07/2019‐06/2021).

**Results:**

This study examined 481 SE episodes in 481 children with a median age of 43 months (1 month to 17 years 11 months), of which 46.2% were female and 50.7% had a previous seizure history. The most frequent acute SE cause was a prolonged, complicated febrile seizure (20.6%). The most common initial seizure types were generalized seizures (49.9%), focal seizures (18.0%), and unknown types (12.1%); 40.5% of patients suffered from refractory SE and 5.0% from super‐refractory SE. The three most common medications administered by nonmedically trained individuals were diazepam, midazolam, and antipyretics. The three most frequent anti‐seizure medications (ASMs) administered by the emergency physician were midazolam, diazepam, and propofol. The three most common ASMs used in the clinical setting were midazolam, levetiracetam, and phenobarbital. New ASMs administered included lacosamide, brivaracetam, perampanel, stiripentol, and eslicarbazepine. Status epilepticus terminated in 16.0% in the preclinical setting, 19.1% in the emergency department, and 58.0% in the PICU; the outcome was unknown for 6.9%. The median PICU stay length was 2 (1–121) days. The median modified Rankin scale was 1 (0–5) on admission and 2 (0–6) at discharge. New neurological deficits after SE were observed in 6.2%. The mortality rate was 3.5%.

**Significance:**

This study provides current real‐world out‐of‐ and in‐hospital data on pediatric SE requiring PICU admission. New ASMs are more frequently used in this population. This knowledge may help generate a more standardized approach.

**Take‐home messages**

This study provides detailed out‐of‐ and in‐hospital data on the etiologies, seizure semiologies, diagnostics, treatments, and outcomes of pediatric status epilepticus episodes requiring pediatric intensive care unit admission.

New anti‐seizure medications are more frequently used in children with status epilepticus requiring pediatric intensive care unit admission.

Despite an overall favorable prognosis, our cohort had substantial morbidity and mortality rates.

## 1 INTRODUCTION

Pediatric status epilepticus (SE) is a common life‐threatening neurological emergency that often requires pediatric intensive care unit (PICU) admission.^1^ The reported frequency for SE is 18–23 per 100 000 children annually. Despite treatment advances over the last two decades, SE continues to be associated with substantial morbidity and mortality. However, controversy exists regarding the extent to which these adverse outcomes are associated with SE and to what extent other factors affect the outcome. Adverse outcomes after SE include cognitive impairment, permanent neurological deficits, hippocampal injury, and subsequent epilepsy.^2^ However, underlying SE etiology appears to play a very crucial role in the outcome.^3^ While the mortality rate is estimated to be 2%–7%,^2^ there is considerable variability in the reported frequencies of adverse morbidity outcomes.

Importantly, rapid SE treatment affects outcomes, and longer treatment delays are associated with poorer outcomes.^4^ Management requires simultaneous resuscitation and medical stabilization, diagnosing the underlying cause, and definitive rapid treatment of both clinical and electrographic seizures. Prompt treatment with benzodiazepines (BZDs) within 5–10 minutes is the first‐line SE treatment. However, many patients require additional treatment with anti‐seizure medications (ASMs), including phenytoin, valproic acid, phenobarbital, and levetiracetam. An up‐to‐date German consensus guideline on SE in children has not yet been published. The publication of such guidelines may improve the timeliness and efficacy of SE treatment.^5^, ^6^, ^7^, ^8^

Since most previously reported epidemiological convulsive SE studies have focused on adult populations, their results might not reliably characterize this disorder in children. Furthermore, data from adults might not directly apply to children since the physical and neurochemical characteristics of the developed brain differ from those of the developing brain. However, several recent studies on children and adolescents with SE, refractory SE (RSE), and super‐refractory SE (SRSE) have been published.^9^, ^10^, ^11^, ^12^, ^13^, ^14^, ^15^, ^16^, ^17^, ^18^

This prospective nationwide surveillance study was performed to provide up‐to‐date real‐world data on the underlying etiologies, diagnostic and therapeutic approaches—both out‐of‐ and in‐hospital—and outcomes of children and adolescents with community‐ or hospital‐onset SE requiring PICU admission for ≥4 hours. In contrast to previous reports, this prospective nationwide study over a 2‐year period included all German children´s hospitals, thus providing a comprehensive and representative data with regard to this important medical issue. The knowledge gained from this study may be informative for the pediatric intensive care and neuropediatric medical communities. It can also be used to implement more standardized approaches with regard to out‐of‐hospital and in‐hospital treatment in this highly susceptible population.

## 2 PATIENTS AND METHODS

This prospective national surveillance study on out‐of‐ and in‐hospital‐onset SE in children and adolescents after the neonatal period requiring admission to a PICU for ≥4 hours was conducted in Germany over 2 years (07/2019‐06/2021). This prospective study was performed according to the principles in the Declaration of Helsinki and with the approval of our Institutional Review Board (University of Saarland, Saarbrücken, Germany; file number: 02/2019). This study was registered at Deutsches Register Klinischer Studien (German Clinical Trials Register; DRKS00016948; July 22, 2019).

Statistical analyses were performed using Microsoft Excel and IBM SPSS Statistics v.25.0 for Windows. Data are presented as means ± standard deviations (SDs) or medians with ranges. Statistical comparisons between groups were performed using chi‐square and Kruskal–Wallis tests. All results with *p* < 0.05 were considered statistically significant.

### 2.1 Definitions

SE was defined according to the International League Against Epilepsy with a duration of >5 minutes for generalized tonic–clonic seizures and >10 minutes for focal seizures, and >10–15 minutes for nonconvulsive SE (NCSE).^19^

Refractory SE was defined as prolonged seizures that continued despite treatment with first‐line BZDs and at least one second‐line non‐BZD ASM.

Super‐refractory SE was defined as SE that continued for ≥24 hours despite anesthetic treatment or recurred when the anesthetic regimen was reduced or withdrawn.

Only the first episode was included in the final data analysis in patients with recurrent SE episodes during the 2‐year study period. However, some patients had previous SE episodes prior to the start of this study.

### 2.2 Diagnostics

Diagnostic procedures were performed during the in‐hospital stay. However, no exact time points for diagnostics, including electroencephalogram (EEG) recordings, were assessed by this study. The SE, RSE, or SRSE diagnosis was made based on the clinical criteria stated above without requiring pathological EEG confirmation. A pre‐existing history of intracerebral lesions, pathologies, and abnormal neurological clinical findings was defined as those occurring ≥6 months prior to SE development. However, the final prior history assessment was at the treating physician's discretion.

Laboratory diagnostics were performed at the treating physician's discretion. They included full blood count; serum chemistry, electrolytes, and glucose levels; inflammatory markers (C‐reactive protein and procalcitonin); ASM drug monitoring; toxicological analyses; and further biomarkers as deemed necessary.

### 2.3 Therapy‐associated side effects

We defined relevant therapy‐associated side effects as any untoward medical occurrence in our study patients that likely had a causal relationship with pharmacological and nonpharmacological treatment modalities based on the treating physician's assessment. They included adverse events that required additional or modified medical treatment, prolonged hospitalization, were potentially life‐threatening, resulted in a persistent or significant disability or incapacity, or resulted in death.

### 2.4 Erhebungseinheit für Seltene Pädiatrische Erkrankungen in Deutschland (ESPED)

The German Pediatric Surveillance Unit (ESPED) was founded in 1992 to provide incidence data and detailed clinical descriptions of rare, childhood‐onset diseases in Germany requiring in‐hospital treatment.^20^ Electronic mailing cards are sent monthly to all children's hospitals in Germany. They ask whether any patients had been newly diagnosed with one of the 12 rare clinical conditions currently under review (active surveillance system; i.e., includes negative reporting [no cases]).^20^ ESPED sends a detailed questionnaire to the reporting hospital in response to a positive answer. Survey periods usually last 2 years. In addition to epidemiological data, further laboratory or genetic testing and imaging modality data can be integrated into individual research projects. Between 1992 and 2017, ESPED completed 96 prospective studies of rare diseases in children.^20^ Therefore, ESPED surveys are an important contributor to the clinical epidemiology of children with rare diseases.^20^

We used the modified Rankin and PEDSS scales to assess disease severity and outcome.^21^, ^22^

*The main research questions assessed:*

1. Underlying etiologies, seizure semiology, and seizure duration.
2. Diagnostic procedures.
3. Out‐of‐ and in‐hospital therapeutic interventions.
4. SE‐related morbidity at discharge and mortality rate

in children and adolescents requiring PICU admission ≥4 hours.

## 3 RESULTS

Three hundred thirty‐six children's hospitals in Germany were contacted during the 2‐year study period, of which 156 enrolled patients for this study. Five hundred eighty cases were initially reported to ESPED. After the removal of all duplicate (n = 17) and false (n = 26) reports, reports containing missing data (n = 48), and reports for patients with recurrent SE during the 2‐year study period (n = 8), 481 episodes in 481 children with SE requiring PICU admission for ≥4 hours were included in the final analysis, of which 222/481 (46.2%) were female and 259/481 (53.8%) were male. Two hundred fifty‐seven (54.4%) SE episodes occurred out‐of‐hospital, and 224 (46.6%) occurred in‐hospital. The median (range) age of the enrolled patients was 43 months (1 month to 17 years 11 months), with 171/481 (35.6%) episodes occurring in infancy (postneonatal to 2 years), 255/481 (53.0%) during childhood (2‐12 years), and 55/481 (11.4%) during adolescence (12‐18 years). A history of prematurity was noted in 30/481 (6.2%) patients.

### 3.1 Etiology

**FIGURE A1:**
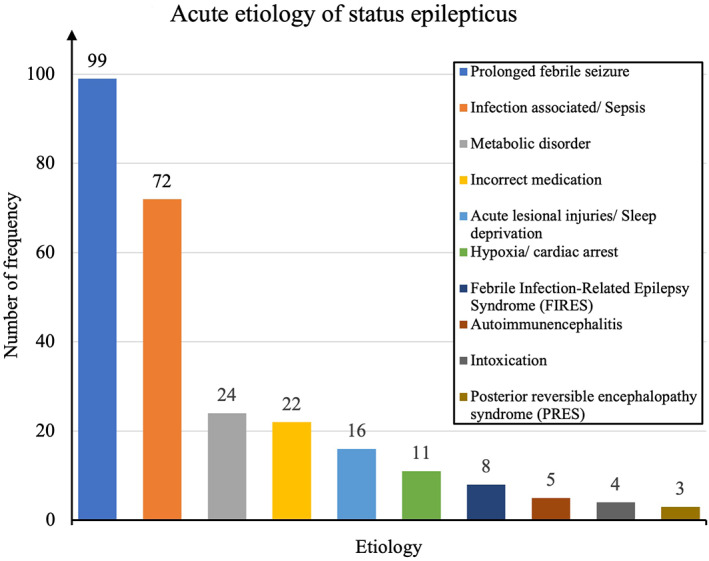
Acute status epilepticus etiology.

The most commonly identified acute SE causes (acute symptomatic: 266/481; 55.3%) are shown in Figure A1, with the three most frequent causes being prolonged febrile seizures (99/481; 20.6%), infection‐associated/sepsis (72/481; 9.8%), and metabolic disorders (24/481; 5.0%). In this study cohort, a pre‐existing history of intracerebral lesions and pathologies and abnormal neurological clinical findings were reported: intracranial hemorrhage (25/481; 5.2%) with posthemorrhagic hydrocephalus (21/481; 4.4%), periventricular leukomalacia (14/481; 2.9%), perinatal asphyxia (14/481; 2.9%), central nervous system (CNS) tumors (11/481; 2.3%), stroke (8/481; 1.7%), nonhemorrhagic hydrocephalus (8/481; 1.7%), cortical dysplasias (2/481; 0.4%), and traumatic brain injury (1/481; 0.2%); remote symptomatic: 104/481 (21.6%).

**FIGURE A2:**
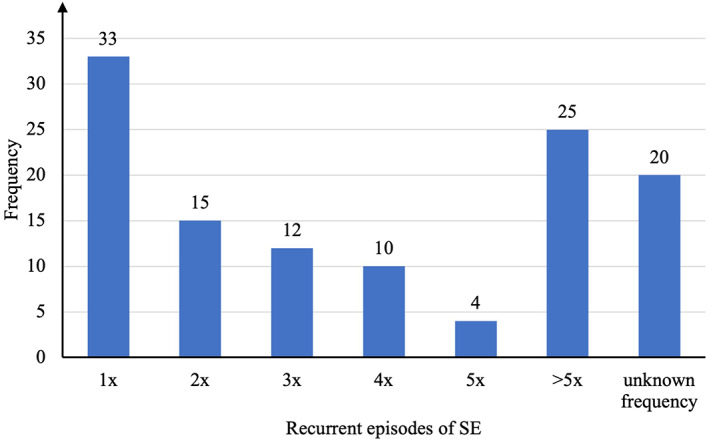
Numbers of patients with previous status epilepticus episodes (n = 119).

A prior epilepsy history was reported for 243/481 (50.5%) patients, with 58/481 (12.1%) patients suffering from genetic epilepsies and syndromes: developmental and epileptic encephalopathies (Dravet syndrome), 21, and infantile epileptic spasm syndrome (West syndrome), 1; Rett syndrome, 5; childhood and juvenile absence epilepsy, 4; Lennox–Gastaut syndrome, 4; Wolf–Hirschhorn syndrome, 4; Angelman syndrome, 4; Tuberous sclerosis complex, 4; self‐limited epilepsy with centrotemporal spikes (Rolandic epilepsy), 3; etiology‐specific syndromes (Sturge–Weber syndrome), 3; self‐limited epilepsy with autonomic seizures (Panayiotopoulos syndrome), 2; epilepsy with myoclonic atonic seizures (Doose syndrome), 1 as well as Ohtahara syndrome; and Watanabe epilepsy, 1 each; defined electroclinical syndromes (38/481 (7.9%)). In 15/481 (3.1%) patients, the etiology was categorized as progressive, and in 58/481 (12.1%) patients, the etiology was categorized as crytogenic. Previous SE episodes were reported for 119/481 (24.7%) patients (Figure A2).

### 3.2 Seizure semiology

The most common initial seizure semiology was generalized in 240/481 (49.9%; 234/240 motor; 4 NCSE) patients and focal in 183/481 (38.0%; 167/183 motor; 8 NCSE) patients, with 72/183 (39.3%) episodes evolving into generalized seizures, and unknown in 58 (12.1%; 27/58 motor) patients.

The mean initial seizure duration was 26.8 minutes (median = 10 minutes [1 minutes to 7.5 hours]) with recurrent epileptic seizures without regaining consciousness in 247/481 (51.4%) episodes. Refractory SE occurred in 195 (40.5%) episodes and SRSE in 24 (5.0%) episodes. The median total SE duration was 90 minutes (5 minutes to 17 days).

### 3.3 Diagnostics

A laboratory diagnostic blood work‐up was performed for 464/481 SE episodes (96.5%), a spinal tap in 244/481 (50.7%), ASM levels in 165/481 (34.3%), and toxicological analyses in 34/481 (7.1%). Amplitude‐integrated or conventional EEGs were performed for 396/481 episodes (82.3%), including 302 pathological EEGs (76.2%). Cerebral magnetic resonance imaging was performed for 229/481 episodes (47.6%; pathological: 124 [25.8%]), cranial computed tomography in 112/481 (23.3%; pathological: 38 [7.9%]), and cerebral sonography in 62/481 (12.8%; pathological: 18 [3.7%]).

Treating physicians stated that they used an internal clinical guideline for 417/481 (86.7%) episodes but did not act according to any specific SE guideline for 64/481 (13.3%).

### 3.4 Treatment

Status epilepticus terminated in 77/481 (16.0%) patients in the out‐of‐hospital setting, 92/481 (19.1%) in the emergency department, and 279/481 (58.0%) in the PICU. Status epilepticus termination location was unknown in 33 (6.9%) patients.

**FIGURE A3:**
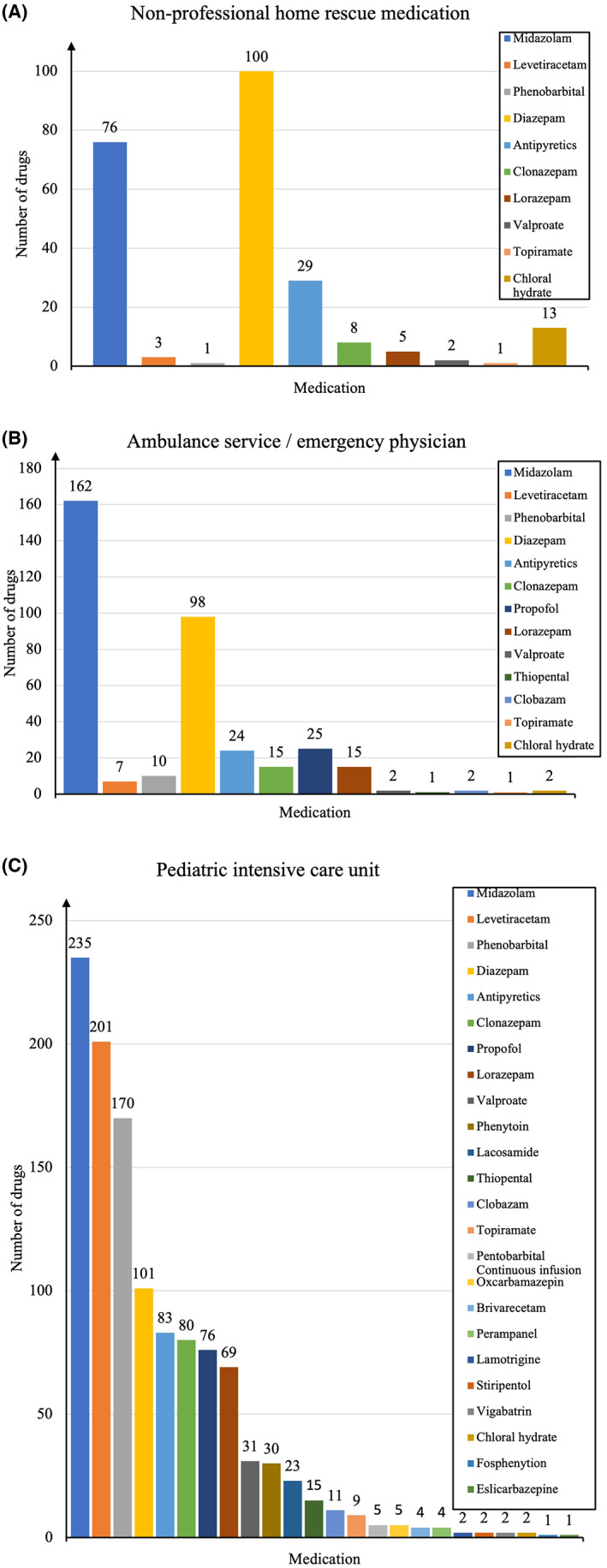
ASM and antipyretic use at home, by the emergency physician, and in the PICU. A: Medications administered by nonmedically trained individuals at home. B: Medications administered by the ambulance service and emergency physicians. C: Medications administered by the PICU.

**FIGURE A4:**
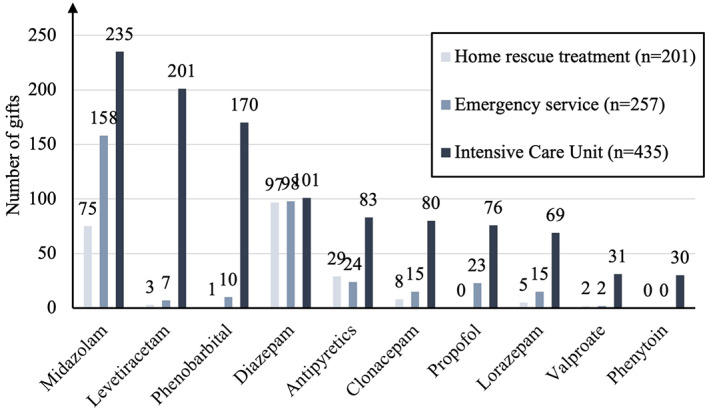
The 10 most frequently used medications by location (out‐of‐hospital, emergency services, and PICU).

Different drugs were widely used in our cohort (Figures A3A–C and A4). The patient initially received home rescue medications administered by nonmedically trained individuals for 201/481 (41.8%) SE episodes. Diazepam was administered most frequently, followed by midazolam. Antipyretics (paracetamol or ibuprofen) were also given (Figure A3A). This therapy included the administration of two or more ASMs in 18/201 (8.9%) patients (median = 0 [0–3]).

Overall, 257/481 (53.4%) patients were treated by the ambulance service and emergency physicians who administered ≥2 ASMs to 74/257 (28.8%) patients (median = 1 [0–5]). The three most common ASMs used by the emergency physician were midazolam, diazepam, and propofol (Figure A3B).

In addition, 435/481 (90.6%) patients were treated with medication in the hospital. The three most common ASMs used in the clinical setting were midazolam, levetiracetam, and phenobarbital (Figure A3C). New ASMs, including lacosamide, brivaracetam, perampanel, stiripentol, and eslicarbazepine, were also used in a few patients. In addition, 312/481 (64.9%) patients received ≥2 different ASMs (median = 2 [0–10]).

During the treatment period, a median of 2 (1–10) ASMs was used in our cohort, with a low rate of ASM‐associated side effects. Figure A4 (The 10 most frequently used medications by location (out‐of‐hospital, emergency services, and PICU)) shows the 10 most frequently used ASMs out‐of‐hospital, in emergency departments, and in‐hospital (PICU). Notably, two patients with RSE were treated with immunotherapy (methylprednisolone). During in‐patient treatment, relevant therapy‐associated side effects were reported for 56/481 (11.6%) patients. The three most common side effects were respiratory insufficiency (ineffective respiratory drive) in 41/56 (73.2%) patients, arterial hypotension in 7/56 (12.5%), and aspiration pneumonia in 4/56 (7.1%). They were thought to be due to both the ASM and SE. Notably, one patient experienced valproate‐induced encephalopathy.

Intubation was performed for 126/481 (26.6%) patients; the median ventilation duration was 15 hours [1–1087 hours]. Intubation was performed for diagnostic studies and treating SE with general anesthesia.

### 3.5 Outcome

**FIGURE A5:**
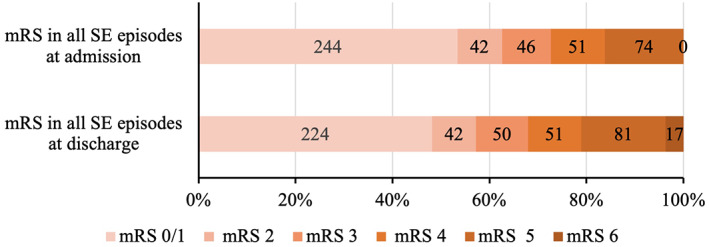
Modified Rankin scale in children with SE before status onset and at discharge (n = 481).

The median (range) length of stay was 2 (1–121) days in the PICU and 5 (1–126) days in the hospital. The mean ± SD modified Rankin scale was 1.85 ± 1.95 (median = 1 [0–5]) on admission (457/481) and 2.12 ± 2.10 (median = 2 [0–6]) at discharge (455/481; Figure A5: Modified Rankin scale in children with SE before status onset and at discharge [*n* = 481]).

The median PEDSS score was 3 (range: 0–6) in survivors and 4 (range: 2–5) in nonsurvivors (*P* < 0.05). New neurological deficits after SE were observed in 30/481 (6.2%) patients. Our study cohort's mortality rate was 3.5% (17/481). While the most frequent cause of death was mitochondriopathy (3/481), the causes were heterogeneous (Table A1).

## 4 DISCUSSION

This large, 2‐year prospective surveillance study provides real‐world, systematic data on underlying etiologies, seizure types, out‐of‐ and in‐hospital diagnostics, and treatment modalities in children with SE requiring PICU admission for ≥4 hours. Consistent with previous reports, febrile convulsive SE was the most common cause in our cohort.^9^, ^10^, ^11^ A pre‐existing history of intracerebral lesions and pathologies and abnormal neurological clinical findings were reported in a substantial proportion in our study cohort, including (in decreasing order) intracranial hemorrhage with posthemorrhagic hydrocephalus, periventricular leukomalacia, perinatal asphyxia, CNS tumors, stroke, nonhemorrhagic hydrocephalus, cortical dysplasias, and traumatic brain injury. Of interest and contrary to our study, another prospective study reported that the most common acute symptomatic etiologies were CNS infection (55%), vascular insult (21%), toxin (8%), electrolyte imbalance (8%), and trauma (8%).^12^

A prior epilepsy history was reported in a high percentage of our patients (50.5%), with a large proportion suffering from underlying genetic epilepsies and syndromes (12.1%). Previous SE episodes were reported for 24.7% of our study cohort. This finding contrasts with previous reports that estimated <15% of children would have a prior epilepsy history.^11^ However, it must also be noted that no precise trigger or underlying etiology could be identified in some patients. In addition, our study included sicker patients requiring PICU admission for ≥4 hours.

Consistent with previous reports, age was the main determinant of SE epidemiology in our cohort. Even within the pediatric population, substantial differences exist in incidence, etiology, and frequency of pre‐existing neurological abnormalities or seizures between younger and older children.^11^ These findings accord with febrile SE occurring mostly in younger children in our study cohort, with a median (range) age of 43 months (1 month to 17 years 11 months). Moreover, 24.7% of patients in our study cohort had recurrent SE episodes compared with 34% in the study by Mitchell et al.^13^

In our study cohort, 40.5% of patients suffered from RSE and 5.0% from SRSE, broadly comparable to those reported by Kravljanac et al. (50.7% and 7.1%, respectively) and Schubert‐Bast et al. (26.8% and 12.8%).^14^, ^15^ Status epilepticus occurrence in children incurred mean treatment costs of €15 880, with €4119 for nonrefractory SE, €13 864 for RSE, and €75 358 for SRSE.^19^ Of note and interest, PICU stay length in our cohort was significantly shorter than in a previous German study.^16^

In addition to epidemiological data, our study provides important insights into the current out‐of‐ and in‐hospital management for diagnostics and treatment. A comprehensive diagnostic work‐up was performed for most of our patients. The American Academy of Neurology guidelines for the diagnostic assessment of a child with convulsive SE reported that abnormal results among children included low ASM levels (32%), neuroimaging abnormalities (8%), electrolytes (6%), inborn errors of metabolism (4.2%), ingestion (3.6%), CNS infections (2.8%), and positive blood cultures (2.5%).^23^ The recent Neurocritical Care Society guidelines indicate that initial etiologic testing should include bedside finger‐stick blood glucose and laboratory testing, including blood glucose, serum electrolytes, complete blood count, a basic metabolic panel, and ASM levels. Depending on the clinical scenario, other diagnostic (e.g., neuroimaging or lumbar puncture) and laboratory (e.g., liver function, coagulation, arterial blood gas, toxicology, and inborn errors of metabolism) tests may be required. Continuous EEG monitoring may also be required for patients who do not wake after their clinical seizures cease.^5^ Unfortunately, based on our data collection and analysis, it was impossible to determine when diagnostic procedures were performed.

Furthermore, our study sheds light on differential medical treatment, such as ASM administration by nonmedically trained individuals, the emergency team, and in‐hospital professional medical staff. The three most common home rescue medications administered by nonmedically trained individuals were diazepam, midazolam, and antipyretics. The three drugs most frequently administered by emergency physicians and teams were midazolam, diazepam, and propofol. The three ASMs most commonly administered in‐hospital were midazolam, levetiracetam, and phenobarbital.

Most hospitals use internal clinical guidelines to treat SE in children and adolescents. However, since 13.1% of treating physicians treated SE without any clinical guidelines, there is an apparent need for implementing evidence‐based guidelines for managing children and adolescents with SE. Treatment guidelines for convulsive SE have previously been directed to treat SE in the hospital. However, since most childhood seizures start in the community, out‐of‐hospital treatment should also play an important role. Management requires simultaneous resuscitation and medical stabilization, diagnosing the underlying cause, and definitive rapid treatment of both clinical and electrographic seizures. While the first‐line SE treatment is prompt BZD administration, many patients will require additional treatment with other ASMs such as phenytoin, valproic acid, phenobarbital, or levetiracetam. Refractory SE is diagnosed when the initial BZD and additional ASM fail to terminate SE, as happened with many patients in our study cohort.

Vasquez et al. showed that pediatric SRSE patients with delayed non‐BZD ASM initiation had more medical complications, higher mortality, and lower return to neurological baseline than non‐SRSE patients. However, these associations were not adjusted for potential confounders.^17^ Delayed BZD use and underdosing were associated with SE refractoriness and unfavorable outcomes.^18^ Unfortunately, our data are not entirely conclusive as to whether ASMs were used appropriately in our study cohort. However, a pattern of repeated BZD use was seen in our study by nonmedically trained individuals, the emergency team, and in‐hospital medical staff.

Various ASMs were used in our study cohort to terminate SE. Sheehan et al.^24^ showed that more than one‐third of patients received >2 BZDs before escalating to a non‐BZD ASM. This finding is generally consistent with Uppal et al., who reported that excessive BZD use and a delay in both definite SE treatments led to escalation from first‐ to second‐line ASMs.^25^ Cavusoglu et al. showed that phenytoin remains one of the most efficient ASMs. They speculated whether a shortened SE episode due to prompt treatment might lead to better outcomes with fewer neurological complications.^26^ In our cohort, phenytoin was administered to 30 children with SE during their in‐hospital treatment phase. Other ASMs exclusively given by emergency physicians or hospital staff included propofol and valproic acid. Levetiracetam was another frequently used ASM in our cohort (Figure A5), with recent reports showing that it was at least as effective as phenytoin in terminating SE in children.^27^, ^28^

New ASMs, including lacosamide, brivaracetam, perampanel, stiripentol, and eslicarbazepine, were used in our study cohort, albeit in fewer patients. The use of diverse ASMs may reflect the clinical condition's severity in our study cohort. However, it may also indicate the lack of a standardized clinical approach. Moreover, there is evidence that early immunotherapy is beneficial in RSE, even when a definite immune etiology has not been identified.^29^ Interestingly, only two patients with RSE in our study cohort were treated with immunotherapy (methylprednisolone).

Contrary to diagnostic procedures, treatment modalities were divided by time course into prehospital (nonmedically trained individuals), emergency services, and in‐hospital, including PICU, in our study, providing a differentiated perspective on this important issue. Notably, SE terminated in 16.0% of patients in the prehospital setting, 19.1% in the emergency department, and 58.0% after PICU admission, reflecting SE severity in our study cohort. Unfortunately, the precise timing of SE termination could not be determined for 6.9%.

The three most common side effects (respiratory compromise, arterial hypotension, and aspiration pneumonia) were thought to be due to ASMs and SE. In our study cohort, intubation was performed for diagnostic studies and treating SE with general anesthesia. It is important to note that propofol was frequently given in our study cohort, but it is not indicated in patients with mitochondrial disease. Notably, one patient with mitochondrial disease who died (patient 11; Table A1) received propofol both out‐of‐ and in‐hospital. Therefore, it is of utmost importance to consider underlying clinical entities when choosing medical and pharmacological treatments in children and adolescents with SE.

While SE is associated with increased morbidity and mortality in children, this appears to be mostly related to the underlying etiology and is less frequent in children than in adults.^1^, ^11^, ^30^ Our study cohort's mortality rate was 3.5%, due to various underlying causes, consistent with a recent meta‐analysis that reported a mortality rate of 3.6%.^9^, ^16^ While all deaths in our study cohort occurred in the SE context, it cannot be stated with absolute certainty that all deaths could be exclusively attributed to SE and its sequelae. Similar to the findings from Tiwari et al.,^22^ mortality was associated with a median PEDSS score ≥ 4 in our study cohort. Kravljanac et al. reported that the main mortality predictors were underlying etiology and pre‐existing neurological abnormalities, while the main morbidity predictor was related to the underlying etiology.^31^ In our study cohort, mortality was associated with severe underlying neurological disease, with mitochondriopathies being the most frequent clinical entity.

New neurological deficits at discharge were seen in 6.2% of patients in our study cohort, substantially lower than previously reported.^3^, ^31^ However, contrary to previous reports, our study design did not entail long‐term clinical follow‐up visits, making a direct comparison difficult. Gaínza‐Lein et al. showed that approximately one‐third of patients without prior epilepsy would develop recurrent unprovoked seizures after the first RSE episode. In addition, 39% of previously normally developing patients will present with new deficits during follow‐up, with longer electroclinical RSE duration being a predictor.^32^ Importantly, some data suggest that using a standardized treatment protocol may be associated with improved outcomes.^33^

Our study had several limitations. First, due to incomplete reporting during the 2‐year study period, not all patients with SE in Germany requiring PICU admission were included. Second, the decision to admit patients to the PICU was at the treating physician's discretion without providing specific clinical reasoning to do so. However, by including only patients with a PICU stay ≥4 hours, the most important reasons for PICU admission were most likely related to severity of SE and treatment modalities, and beyond mere intensive care observation. Nevertheless, while we intended to include only severely affected children, the inclusion criterion of ≥4 hours in PICU appears somewhat arbitrary. Moreover, in our survey, we did not relate the number of SE admissions to other clinical entities requiring PICU admission (e.g., respiratory and trauma). Third, although we were able to demonstrate that in our cohort new ASDs were used, we did not specifically address the timing and dosing of new ASDs. Therefore, the role and efficacy of new ASDs in the treatment of SE in children remain subject to further clinical investigations. Fourth, we did not perform long‐term follow‐up visits with our study cohort.

In summary, our large prospective nationwide study of children and adolescents with SE requiring PICU admission identified heterogeneous underlying SE causes with an overall favorable neurological outcome and survival prognosis. Our study provided a current, differentiated, and detailed real‐world description of SE etiology, diagnosis, and out‐of‐ and in‐hospital treatment and outcome. In addition, we noted differences in the pattern of ASM use, with some patients receiving new ASMs, including levetiracetam, lacosamide, brivaracetam, perampanel, stiripentol, and eslicarbazepine. The publication of guidelines may improve SE treatment timeliness and efficacy.^8^ We believe that our real‐world data will be useful in developing and publishing national and international guidelines for children and adolescents with SE, enabling a standardized approach to pediatric SE diagnosis and treatment to improve outcomes in this vulnerable population.

## AUTHOR CONTRIBUTIONS

Sascha Meyer was the chief investigator and was responsible for study design, data acquisition and analysis, and manuscript writing. Jaro Langer was responsible for data acquisition and analysis and manuscript writing. Stefan Wagenpfeil was responsible for statistical data analysis. Martin Poryo was responsible for manuscript drafting and critical revision. Johannes Goaliath Bay was responsible for manuscript drafting and critical revision. Adam Strzelczyk was responsible for the study design and critical manuscript review. Beate Heinrich was responsible for data compilation. Holger Nunold was responsible for data analysis and processing. Daniel Ebrahimi‐Fakhari was the co‐chief investigator and was responsible for study design, data acquisition and analysis, and manuscript writing.

## CONFLICT OF INTEREST

This study received financial grants from the Dr. Wolf Epilepsy Project and Union Chimique Belge (UCB). Neither the Dr. Wolf Epilepsy Project nor UCB influenced the study design, data compilation, analysis, or presentation. Sascha Meyer reports research grants from Deutsche Forschungsgemeinschaft (DFG), UCB, Novartis, and the Dr. Wolf Epilepsy Project. Jaro Langer, Martin Poryo, Johannes Bay, Stefan Wagenpfeil, Beate Heinrich, and Holger Nunold report no financial support or conflicts of interest. Adam Strzelczyk reports personal fees and grants from Arvelle Therapeutics, Desitin Arzneimittel, Eisai, GW Pharmaceuticals, LivaNova, Marinus Pharma, Medtronic, UCB, and Zogenixs. Daniel Ebrahimi‐Fakhari reports research and travel grants from Tuberöse Sklerose Deutschland e.V. and personal fees from GW Pharmaceuticals.

## ETHICAL APPROVAL

We confirm that we have read the journal's (Epilepsia Open) position on issues involved in ethical publication and affirm that this report is consistent with those guidelines. This study was approved by our local ethics committee (Ethikkommission des Saarlandes, EK Saarbrücken, Germany; file number: 02/2019).

## APPENDIX A

**TABLE A1:** Characteristics of patients with fatal outcomes

Patient	Cause of death	Initial seizure semiology	Rankin before hospitalization	RSE	SRSE
1	Drowning	Generalized	0	Yes	Yes
2	Mitochondriopathy	n/a	n/a	Yes	Yes
3	Posterior reversible encephalopathy syndrome/CMV and HHV‐6 encephalitis in acute myeloid leukemia	Generalized	2	No	No
4	HSV encephalitis	Focal	3	No	No
5	Cerebral edema	n/a	4	Yes	No
6	Encephalitis in carnitine deficiency	Generalized	3	No	No
7	Sepsis in dystroglycanopathy	Focal	5	Yes	Yes
8	Stroke associated with Influenza A infection	Focal	0	Yes	No
9	Mitochondriopathy (Leigh syndrome)	Generalized	5	Yes	No
10	Cardiac arrest	Focal	0	Yes	Yes
11^a^	Mitochondriopathy	Focal	2	Yes	Yes
12	Febrile‐infection‐related epilepsy syndrome (FIRES)	Generalized	0	Yes	Yes
13	Metastatic neuroblastoma	Focal	1	Yes	No
14	Diabetic ketoacidosis	Focal	3	Yes	Yes
15	Acute necrotizing encephalopathy	Focal	0	No	No
16	Nonketotic hyperglycinemia	n/a	5	Yes	Yes
17	Abusive head trauma	Generalized	0	n/a	n/a

Abbreviations: n/a, not applicable; RSE, refractory status epilepticus; SRSE, super‐refractory status epilepticus.

^a^ Note that patient 11 with mitochondriopathy received propofol both out‐of‐ and in‐hospital.

## APPENDIX B

### B.1 Collaborators

Alchikh, Maren; Klinik für Kinder‐ und Jugendmedizin, Helios Klinikum Bad Saarow, Bad Saarow

Aukschun, Ute; University Children's Hospital Freiburg, Freiburg

Bellm, Aliyah; Zentrum für Kinder‐ und Jugendmedizin, Helios Klinikum Krefeld, Krefeld

Blümlein, Ulrike; University Children's Hospital Cottbus, Cottbus

Buss, Michael; Kinderklinik Rosenheim, Rosenheim

Daniels, Anna; University Children's Hospital Essen, Essen

Della Marina, Adela; University Children's Hospital Essen, Essen

Eichholz, Stephan; Kinderklinik Städtisches Klinikum Dresden, Dresden

Eilers, Miriam; Klinik für Kinder‐ und Jugendmedizin, Oberschwabenklinik, Ravensburg

Fransse, Meike; St. Franziskus‐Hospital, Münster

Gohar, Faekah; Klinik für Kinder‐ und Jugendmedizin, Clemenshospital, Münster

Grinstein, Lev; University Children's Hospital Hamburg, Hamburg

Hachmann, Wiebke; Kinderklinik Städtische Kliniken Mönchengladbach, Mönchengladbach

Harmsen, Stefani; University Children's Hospital Düsseldorf, Düsseldorf

Herberger, Sarah; University Children's Hospital Saarland, Homburg

Heußinger, Nicole; Children's Hospital Nürnberg, Nürnberg

Kaluza, Elke, Kinderklinik Kaiserslautern, Kaiserslautern

Koppitz, Martin; Sana Kinderklinik Offenbach, Offenbach

Kulka, Remy; University Children's Hospital Halle, Halle

Lange, Mario; University Children's Hospital Regensburg, Regensburg

Leinert, Johann; University Children's Hospital Mannheim, Mannheim

Lörinczy, Emese; Klinikverbund Südwest, Kinderklinik Böblingen

Müller, Philipp; University Hospital Freiburg, Freiburg

Neusser, Sophia; Klinik für Kinder‐ und Jugendmedizin, Oberschwabenklinik, Ravensburg

Pareja Romain, Beatriz; Kinderklinik Ludwigsburg, Ludwigsburg

Pietsch, Martin; Deutsches Rotes Kreuz‐Kinderklinik Siegen, Siegen

Propson, Sven; Children's Hospital Leverkusen, Leverkusen

Renk, Hanna; University Children's Hospital Tübingen, Tübingen

Reutershahn, Elke; Kinderklinik, Helios St. Johannes Klinik Duisburg, Duisburg

Schmid, Simon; University Children's Hospital Tübingen, Tübingen

Schmitt, Sabrina; Mutterhaus der Borromäerinnen, Kinderklinik Trier, Trier

Tschiedel, Eva; University Children's Hospital Essen, Essen

Thomas, Lukas; Darmstädter Kinderkliniken, Darmstadt

Vilserm, Constanze; St. Georg Klinikum, Klinik für Kinder‐ und Jugendmedizin, Leipzig

Vorbrodt, Katja; University Children's Hospital Magdeburg, Magdeburg

Wiebe, Beatrix; Klinik Asklepios Klinik Sankt Augustin, Bonn

Wördehoff, Rosa; Kinderklinik Itzehohe, Itzehoe

All collaborators from Germany.
